# Objective assessment of tapering of the fingers in adults

**DOI:** 10.1371/journal.pone.0279202

**Published:** 2022-12-28

**Authors:** Seth M. Weinberg

**Affiliations:** 1 Center for Craniofacial and Dental Genetics, Department of Oral and Craniofacial Sciences, University of Pittsburgh, Pittsburgh, PA, United States of America; 2 Department of Human Genetics, University of Pittsburgh, Pittsburgh, PA, United States of America; 3 Department of Anthropology, University of Pittsburgh, Pittsburgh, PA, United States of America; Ohio State University, UNITED STATES

## Abstract

Mild proximal-to-distal tapering of the fingers is a relatively common trait in humans. When more extreme, finger tapering is a feature observed in many genetic syndromes. The range of variation for finger tapering in the general population is not well understood, and sex differences in the degree of tapering, while suspected, have not been documented. Part of the difficulty is a lack of objective methods to evaluate finger tapering. In the present report, we developed a tapering index based on linear measures derived from digital hand scans. We measured this index in a sample of 166 male and 166 age-matched female adults. We then looked at correlations both among fingers and with demographic and anthropometric variables, followed by tests for sex differences. We observed weak correlations between tapering and age, height and weight. Correlations between pairs of fingers tended to be more in the moderate range and were highest among the middle three fingers (ranging from 0.34 to 0.64). Tapering tended to increase slightly moving radially across the hand from the fifth finger to the second finger. Males showed less tapering than females for all fingers, with statistically significant differences involving the left second finger (p = 0.003), left fifth finger (p< 0.001), right second finger (p = 0.038), and right fourth finger (p = 0.021). Finally, we established baseline norms for both males and females out to three standard deviations. Our results indicate that finger tapering is present, to some degree, in most of the population and that the trait can be measured using a relatively simple and non-invasive method. These findings may have relevance for fields as diverse as medical genetics, forensics, and industrial design.

## Introduction

Tapering of the fingers (or digits) is defined as “(t)he gradual reduction in girth of the digit from proximal to distal” [1]. Tapering can impact specific digits or can occur on all digits uniformly. The assessment by the dysmorphologist is through visual inspection of the hands and is therefore considered subjective. In its more extreme forms, digit tapering has been associated with many syndromes (e.g., Coffin-Lowry syndrome; OMIM: 303600) [2]. Less extreme cases, however, can be difficult to assess due to the continuous nature of the trait. Tapering to some degree is present in the general population. Throughout history, tapered fingers have even been considered an aesthetically desirable feature; for example, in the nineteenth century, numerous devices were patented and marketed to women to help them achieve tapered fingertips [3]. However, the degree of finger tapering in the general population has not been formally studied. As a result, we know very little about the normal range of variation for this feature. This has potential implications for the use of finger tapering in clinical genetics, where it can be a challenge to discern whether an anatomical feature is truly dysmorphic or merely atypical. Because finger tapering is a continuous morphological feature, it can be measured and upper and lower bounds can be established statistically. In the present report, we devised a simple method to quantify finger tapering based on digital hand scans. We report descriptive statistics for a sample of U.S. adults, look at associations with factors like age and body size, and compare the average tapering between males and females.

## Materials and methods

The sample in this study was recruited as part of a large genetic epidemiological project of nonsyndromic orofacial clefting targeting individuals in the western Pennsylvania region. As part of the deep phenotyping protocol for that project, images of the hands were collected from participants (described in further detail below). The present sample is focused on adults of recent (self-identified) European ancestry, recruited as either the unaffected relatives of index cases with an orofacial cleft or as unaffected controls. Starting with an initial sample of 754, individuals were excluded if they (1) were affected with a cleft of the lip and/or palate; (2) had missing data; (3) had any reported hand malformations (e.g., syndactyly) or conspicuous trauma or injuries; (4) reported having a genetic syndrome; (5) were over 60 years of age; or (6) reported an ancestry other than European. The upper age cut off was applied to mitigate the potential for age-related joint changes to impact the results [4]. The ancestry exclusion was applied due to the small number of non-European participants available in the dataset, which precluded a proper evaluation of the impact of ancestry on measurement outcomes. Based on these criteria, a total of 218 participants were excluded from further analysis. This resulted in a study sample of 166 adult males, to which an equal number of adult females (n = 166) were matched out of a total pool of 370 eligible females. The matching was based on age to within one year. In our final study sample, the mean age of males was 34.11 years (sd = 12.68) and the mean age of females was 34.16 years (sd = 12.57), indicating that the one-to-one matching was very tight (t = -0.036; df = 330; p = 0.971). All participants provided written informed consent prior to participation and all work was approved by the Human Research Protection Office (protocol: STUDY19080127) at the University of Pittsburgh.

**Fig 1:**
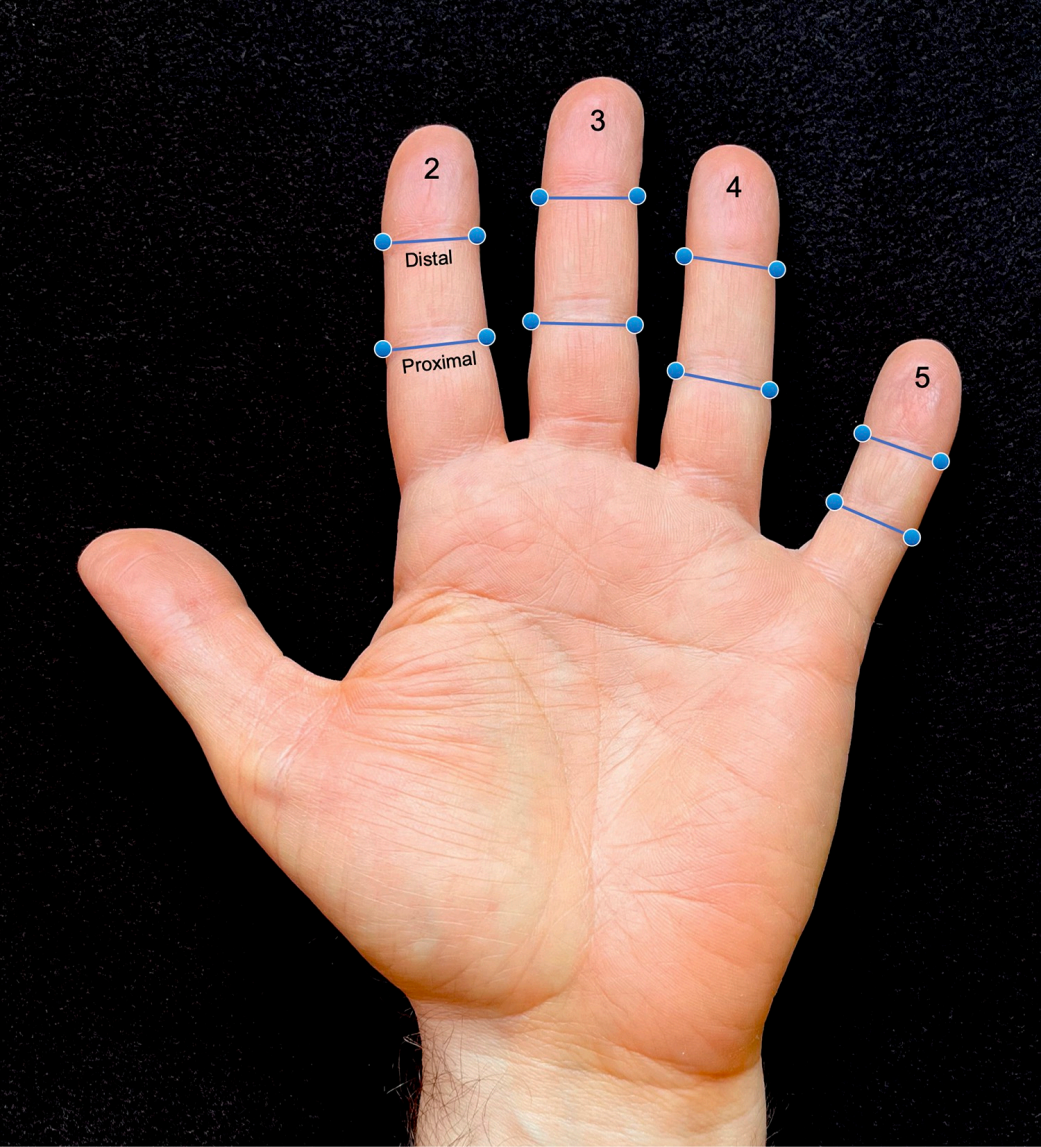
Image showing landmarks and distances used to quantify finger tapering. Landmarks were placed at the radial and ulnar margins of the creases corresponding to the proximal and distal inter-phalangeal joints of fingers 2–5. From these landmarks, finger widths were calculated at the proximal and distal joints on each finger on each hand. The tapering index was then calculated by dividing the distal inter-phalangeal joint width by the proximal inter-phalangeal joint width for each finger. Measures were not collected on the thumb (first finger). Identical measures were collected on the right hand (not shown here).

High-resolution digital hand scans were collected on each participant with a large format flat-bed scanner (Epson GT-20000, Nagano, Japan). Participants were instructed to place their hands palm down on the glass with light pressure and their fingers extended and separated in a natural, unstrained posture. Once acquired, the operator immediately reviewed the quality of the scan, inspecting for potential problems related to excess pressure, movement, or posture. Scans were re-acquired as necessary. The digital scans were then imported into the program tpsDIG2 (http://sbmorphometrics.org/index.html) and 32 landmarks (16 per hand) were placed manually at the visible radial and ulnar margins of the creases corresponding to the proximal and distal inter-phalangeal joints of fingers 2–5 (Fig 1). The choice of landmarks was guided by two factors: (1) ease of identification due to the presence of inter-phalangeal flexion creases to help guide placement and (2) the fact that the skin at the lateral margin of the inter-phalangeal joints is very close to the underlying joint capsule, making this anatomical region resistant to deformation due to pressure. It was not possible to capture data on the thumb because it was slightly rotated with the hand in the aforementioned posture. Similar landmarks collected from 2D digital scans used in a prior study of finger measurements showed very low error from repeated measures, with intraclass correlation coefficients exceeding 0.97 [5]. Using *x*,*y* coordinates from the collected landmarks, finger widths were calculated at the proximal and distal joints of fingers 2–5 on each hand (Fig 1). A tapering index was then calculated by dividing the distal inter-phalangeal joint width by the proximal inter-phalangeal joint width for each finger. A tapering index value of 1.00 would indicate the width of the finger was uniform with no tapering. Any value below 1.00 indicates some degree of tapering; the lower the index the more tapered the finger.

In addition to providing descriptive statistics, we examined the correlation between tapering and demographic or anthropometric factors (age, height, and weight). Since distributions for the tapering index were normal based on Kolmogorov-Smirnov and Shapiro-Wilk tests (all p > 0.05), we used standard Pearson correlations. We then compared mean tapering between our age-matched males and females with two-sample t-tests (one-tailed) to investigate the hypothesis that on average females have more tapered fingers. The nominal significance threshold was set at 0.05, but we also report a more stringent threshold after adjusting for the number of traits tested (0.05/8 = 0.006). In addition, we report the Cohen’s d effect size to help interpret the magnitude of the male-female differences.

## Results

The finger taper index generally showed a weak positive correlation with age (i.e., tapering was reduced as age increased) in both males and females (Table 1). There was very little correlation with height, with males showing mostly weak positive relationships and females showing weak negative relationships. None of the correlations with height were statistically significant.

**Table 1:** Correlations for tapering among fingers and between fingers and other variables.

	Age	Height	Weight	Finger 2 (L)	Finger 3 (L)	Finger 4 (L)	Finger 5 (L)	Finger 2 (R)	Finger 3 (R)	Finger 4 (R)	Finger 5 (R)
Age		-	-	.07	.04	**.25**	-.10	.20	.11	**.23**	.06
Height	-		-	-.05	-.10	-.15	.01	-.14	-.12	-.07	-.03
Weight	-	-		**-.16**	-.04	-.12	-.06	-.14	-.11	-.10	-.05
Finger 2 (L)	.12	.02	**-.20**		**.50**	**.37**	**.24**	**.56**	**.43**	**.37**	**.21**
Finger 3 (L)	**.20**	.08	**.18**	**.48**		**.50**	**.33**	**.46**	**.56**	**.43**	**.36**
Finger 4 (L)	.11	.08	.12	**.45**	**.64**		**.26**	**.40**	**.43**	**.64**	**.21**
Finger 5 (L)	.11	.07	.04	**.33**	**.33**	**.43**		**.23**	.14	.11	**.49**
Finger 2 (R)	**.28**	.01	-.09	**.48**	**.40**	**.41**	**.29**		**.44**	**.34**	**.30**
Finger 3 (R)	**.24**	-.07	.04	**.49**	**.65**	**.56**	**.36**	**.53**		**.49**	**.26**
Finger 4 (R)	**.22**	-.01	.05	**.46**	**.55**	**.59**	**.41**	**.40**	**.61**		**.23**
Finger 5 (R)	**.21**	.11	.06	**.25**	**.31**	**.31**	**.57**	**.28**	**.36**	**.39**	

Correlations below the diagonal for males and above the diagonal for females. Correlations in bold face indicate that the correlation was statistically significant at p ≤ 0.05. Age measured in years. Height measured in cm. Weight measured in lbs.

A similar pattern (males weak positive and females weak negative) was observed for weight. These correlations were statistically significant in the few instances where the correlation approached +/- 0.20 (e.g., left 2^nd^ finger).

Looking at the relationship across fingers, most show weak-to-moderate positive correlations (Table 1). Fingers near one another tended to show higher correlations, compared to fingers further apart. The highest correlations tended to involve the 3^rd^ and 4^th^ fingers. Moreover, the corresponding fingers on the left and right hands tended to show higher correlations with one another than with other fingers. The pervasive positive correlations among fingers suggests the tapering pattern impacts the fingers as a group, rather than affecting isolated fingers.

For all fingers, tapering was decreased (index was higher) in males. This effect was nominally significant (p < 0.05) for four of the eight fingers tested: left 2^nd^ finger, left 5^th^ finger, right 2^nd^ finger, and right 4^th^ finger (Table 2). The left 3^rd^ and 4^th^ fingers were suggestive (p > 0.10). Both the left 2^nd^ finger and left 5^th^ finger also exceeded the more stringent Bonferroni threshold. Because weight showed a significant relationship with the tapering index for some fingers, we also repeated these sex comparisons after removing the effects of weight using an ANCOVA design. This confirmed the original results. The effect sizes for these sex differences tended to be small, except for the left 5^th^ digit which showed a moderate effect (d = 0.51).

**Table 2:** Sex differences in finger tapering in U.S. adults.

	Males (n = 166)		Females (n = 166)		t-test	ANCOVA	Effect Size
	Mean	sd	Mean	sd	p	p	d
Finger 2 (L)	.856	.029	.847	.029	**.003**	**< .001**	.31
Finger 3 (L)	.860	.036	.855	.029	.086	.219	.15
Finger 4 (L)	.864	.043	.858	.037	.093	.125	.15
Finger 5 (L)	.913	.042	.894	.032	**< .001**	**< .001**	.51
Finger 2 (R)	.864	.031	.858	.030	.038	.009	.20
Finger 3 (R)	.856	.034	.853	.031	.199	.162	.09
Finger 4 (R)	.870	.037	.862	.035	.021	.020	.22
Finger 5 (R)	.899	.038	.894	.035	.111	.140	.14

Two-sample t-test p-value (one-tailed); ANCOVA p-value (one-tailed), adjusting for weight; d = Cohen’s d effect size. A positive value for d indicates that females had more tapering than males. Bold face in the p-value column indicates that the value exceeds Bonferroni correction threshold. L = left. R = right.

Across all fingers, tapering index values ranged from a low of 0.733 to a high of 1.04. Only four individuals had values that exceeded 1, but only by the slightest degree; these were all males, ranging in age from 20 to 48.6 years, and all instances were limited to the left 5^th^ finger. This small number of extreme values could have impacted the large sex difference observed for this finger. Tapering tended to increase (index was lower) moving from 5^th^ to the 2^nd^ finger; this was observed in both hands and for both sexes (Table 2). Sex specific norms for each finger out to three standard deviations are available in S1 Table. The complete raw data for all 332 individuals is provided in a S1 File.

## Discussion

In this report, we introduced a relatively simple method to quantify tapering for the 2^nd^– 5^th^ fingers. Using 2D digital hand scans, we calculated a tapering index based on the width of the finger at two regions: one more proximal and one more distal. By measuring this index on a large age-matched sample of adult males and females, we established sex-specific norms for finger tapering and investigated sex differences. The summary statistics we generated can be used to calculate z-scores for individuals matching the demographic background of the samples used here.

We found that almost all individuals in our cohort showed some degree of tapering, with males consistently showing less tapered fingers than females on average, confirming our initial hypothesis. However, the magnitude of the sex difference was not uniform across the fingers and was most pronounced on the left and right 2^nd^ fingers, the left 5^th^ finger, and the right 4^th^ finger. Looking within males and females separately, we observed a weak positive correlation with age, indicating that tapering tended to decrease slightly as individuals got older. We found little evidence of association with height, but some association with weight, with males mostly showing weak positive correlations and females showing weak negative correlations. It is not clear what might be driving this opposing weight effect, but the correlations were very small and mostly non-significant. The more moderate positive correlations observed between different sets of fingers suggests that an individual’s tendency toward tapering (or non-tapering) was likely to impact multiple fingers on both hands. However, we found that the correlation was weakest between the 5^th^ finger and the other three fingers. Interestingly, a similar phenomenon has been reported for fingerprints; i.e., the “pattern block” showing the highest correlations among the middle three fingers [6]. We also found that tapering tended to increase slightly moving from the 5^th^ finger to the 2^nd^ finger. The biological mechanisms underlying this pattern are unknown, but a reasonable point of departure might be the molecular signaling pathways that provide axial identity to the developing distal limb [7].

The objective measurement approach described here has the potential to improve the phenotypic assessment of limb dysmorphology [1], which currently involves a great deal of subjective judgement. This is particularly relevant in the context of medical genetics, where syndrome assignment can present a significant challenge. For example, the reference values generated here could aid diagnoses by establishing whether an individual’s finger tapering falls within or outside of the bounds of normal variation. Moreover, the general approach and norms described here may also serve as a reference for reconstructive hand surgery [8, 9], aid in forensic investigations [10, 11], and inform design and ergonomic applications [12, 13]. For example, sex determination from the hand and digits can help with the identification of partial remains. Our results indicate that digit tapering is a sexually dimorphic characteristic and, as such, may be useful in such forensic applications.

The present analysis was limited to adults of recent European ancestry. Thus, the applicability of these results to individuals from other ancestral groups or to sub-adults cannot be assumed. Establishing quantitative patterns for hand and finger traits, such as tapering, in children is important since many syndromes are first recognized at an early age. The inability to accurately capture width dimensions on the thumb is another limitation of this study, resulting from our scanning method. This could potentially be overcome by collecting additional scans with the hand in a different orientation. Lastly, when comparing against the norms generated from this study, care should be taken ensure that compatible measurement methods are used. This is because digit widths measured using alternative techniques, such as 3D scanning and direct anthropometry, could result in systematic differences.

## Supporting information

S1 Table Norms for finger/digit tapering in U.S. adults of European ancestry. (PDF)

S1 File Complete individual-level data used in the analysis. (XLSX)
