# Supplementary material for: Objective assessment of tapering of the fingers in adults
Source: PLoS One. 2022 Dec 28;17(12):e0279202. doi: 10.1371/journal.pone.0279202 (PMC9797067; doi:10.1371/journal.pone.0279202)
Supplement: S1 Table — (PDF) [file pone.0279202.s001.pdf]

S1 Table. Norms for Finger/Digit Tapering in U.S. Adults of European Ancestry.

	Males (n = 166)							Females (n = 166)						
	-3sd	-2sd	-1sd	Mean	+1sd	+2sd	+3sd	-3sd	-2sd	-1sd	Mean	+1sd	+2sd	+3sd
Digit 2 (Left)	0.769	0.798	0.827	0.856	0.885	0.914	0.943	0.760	0.789	0.818	0.847	0.876	0.905	0.934
Digit 3 (Left)	0.752	0.788	0.824	0.860	0.896	0.932	0.968	0.768	0.797	0.826	0.855	0.884	0.913	0.942
Digit 4 (Left)	0.735	0.778	0.821	0.864	0.907	0.950	0.993	0.747	0.784	0.821	0.858	0.895	0.932	0.969
Digit 5 (Left)	0.787	0.829	0.871	0.913	0.955	0.997	1.039	0.798	0.830	0.862	0.894	0.926	0.958	0.990
Digit 2 (Right)	0.771	0.802	0.833	0.864	0.895	0.926	0.957	0.768	0.798	0.828	0.858	0.888	0.918	0.948
Digit 3 (Right)	0.754	0.788	0.822	0.856	0.890	0.924	0.958	0.760	0.791	0.822	0.853	0.884	0.915	0.946
Digit 4 (Right)	0.759	0.796	0.833	0.870	0.907	0.944	0.981	0.757	0.792	0.827	0.862	0.897	0.932	0.967
Digit 5 (Right)	0.785	0.823	0.861	0.899	0.937	0.975	1.013	0.789	0.824	0.859	0.894	0.929	0.964	0.999
